# Miocene orographic uplift forces rapid hydrological change in the southern central Andes

**DOI:** 10.1038/srep35678

**Published:** 2016-10-21

**Authors:** Alexander Rohrmann, Dirk Sachse, Andreas Mulch, Heiko Pingel, Stefanie Tofelde, Ricardo N. Alonso, Manfred R. Strecker

**Affiliations:** 1 Institut für Erd- und Umweltwissenschaften, Universität Potsdam, 14476 Potsdam, Germany; 2 GFZ German Research Centre for Geosciences, Section 5.1: Geomorphology, Telegrafenberg, 14473 Potsdam, Germany; 3 Senckenberg Biodiversity and Climate Research Centre (BiK-F), 60325 Frankfurt/Main, Germany; 4 Institut für Geowissenschaften, Goethe Universität Frankfurt, 60438 Frankfurt/Main, Germany; 5 Departamento de Geología, Universidad Nacíonal de Salta, Conicet, 4400 Salta, Argentina

## Abstract

Rainfall in the central Andes associated with the South American Monsoon and the South American Low-Level Jet results from orographic effects on atmospheric circulation exerted by the Andean Plateau and the Eastern Cordillera. However, despite its importance for South American climate, no reliable records exist that allow decoding the evolution of thresholds and interactions between Andean topography and atmospheric circulation, especially regarding the onset of humid conditions in the inherently dry southern central Andes. Here, we employ multi-proxy isotope data of lipid biomarkers, pedogenic carbonates and volcanic glass from the Eastern Cordillera of NW Argentina and present the first long-term evapotranspiration record. We find that regional eco-hydrology and vegetation changes are associated with initiation of moisture transport via the South American Low-Level Jet at 7.6 Ma, and subsequent lateral growth of the orogen at 6.5 Ma. Our results highlight that topographically induced changes in atmospheric circulation patterns, not global climate change, were responsible for late Miocene environmental change in this part of the southern hemisphere. This suggests that mountain building over time fundamentally controlled habitat evolution along the central Andes.

With average elevations of ~4 km, low internal relief, closed and partially coalesced sedimentary basins, the Andean Plateau (AP, Altiplano-Puna) constitutes the world’s second largest orogenic plateau^1^ (Fig. 1). Topographic growth of the AP and its high eastern margin causes the intercept of moisture-bearing easterly winds, resulting in an efficient orographic barrier with steep E-W rainfall and vegetation gradients^2^,^3^,^4^ (Fig. 1). Today, the undeformed foreland and the eastern flanks of the AP between 13° and 30°S receive summer precipitation of up to 3 m/yr mainly via the southward transport of Amazonian moisture by the South American Low-Level Jet (LLJ) of the South American Monsoon (SAM)^5^. In contrast, leeward basins to the west of this orographic barrier are arid and receive rainfall amounts of less than 0.2 m/yr^2^. These conditions are mirrored by dense C_3_ vegetation on the eastern flanks of the AP, whereas the orogen interior hosts a mixed C_3_/C_4_ vegetation, reflecting aridity^6^.

Stable isotope data suggest that the establishment of present-day climatic conditions, topography, and vegetation patterns in the Bolivian Andes (20–22°S) was coeval with the onset of the LLJ at ca. 9 Ma^7^,^8^. This has been linked to surface uplift of the central AP and redirection of air-mass trajectories in South America^4^,^8^,^9^,^10^. However, discontinuous uplift^11^,^12^, long-term surface evaporation^13^, global climate change^14^, and changes in the water stable isotope compositions occur simultaneously with surface uplift in the Andes^15^. Recent paleotopographic reconstructions challenge these interpretations and postulate the existence of a high-elevation AP in NW Argentina already during the Oligocene^16^,^17^. To evaluate these inconsistencies additional information on the topographic growth and climate south of 22°S is needed, especially in NW Argentina. Previous studies suggest that global climate change did not have a profound affect on South American climate during the time period in question, but rather that the growth of Andean topography reinforced the preexisting climatic regime and promoted further aridification within the AP and along the Pacific cost^18^,^19^. This particularly applies to surface uplift during the establishment of the LLJ and associated reconfiguration in the provision of nutrients, biodiversity, fluvial networks, and other eco-hydrological changes that affected sediment flux toward the foreland and ocean basins^20^.

One commonly encountered impediment in stable isotope paleoaltimetry is the potential transience of stable isotope lapse rates through time, e.g. changes in moisture sources and trajectories due to topographic uplift or climate change^10^,^21^. In this study such transients are considered to be of minor relevance for the intermontane Angastaco Basin (AB) of NW Argentina, as it is located within the Eastern Cordillera (Fig. 1) immediately east of the AP, with former sediment transport directions pointing to unrestricted eastward transport^22^ and no indication for modifications of airflow trajectories due to topographic uplift or changes in the moisture source^2^,^8^,^10^. Prior to deformation and surface uplift the basin was an integral part of the low-elevation (<500 m) foreland^22^. In our study we use a multi-isotope approach combining inorganic and organic proxies to decode moisture supply changes to the AB that reflect evolving eco-hydrological conditions along the AP flanks. Today the basin is located at ca. 2 km elevation and hosts up to 6 km of sediments, deposited during tectonic deformation along the eastern flank of the AP beginning at ca. 9 Ma. Geochronology of abundant intercalated volcanic ashes in the AB furnishes a unique, well-dated record of interlayered organic-rich beds and carbonate-bearing paleosols (Fig. 2; Fig. S1). The former contain lipid biomarkers, specifically *n*-C_27_ to *n-*C_33_ alkanes derived from leaf waxes of higher terrestrial plants that originated in the basin catchment along the eastern AP margin (Fig. 1; Figs S2–7 and 10; Table S1)^23^. We focus on paleoenvironmental proxy data from the stable isotopic composition of pedogenic carbonates (δ^18^O_sc_), volcanic glass (δD_vg_), and *n*-C_29_ and *n*-C_31_ alkanes (δD_wax_) (see SI)^24^. The δD_wax_ signal typically forms during the early season as leaves flush over variable timescales. This corresponds to weeks in deciduous plants^25^,^26^ to months in evergreen plants^27^ and coincides with the monsoonal rainy season in the study area. In the southern central Andes δ^18^O_sc_ have also been reported to form during the rainy season, or at its end^28^,^29^. Therefore, both proxies record similar seasonal timeframes (i.e. the austral summer rainy season). We use δD_wax_ as a proxy for the leaf water hydrogen isotopic composition (a function of plant transpiration), soil-carbonate δ^18^O_sc_ as a proxy for soil water δ^18^O values (a function of soil evaporation), and volcanic glass δD_vg_ as a proxy for precipitation δD values, and their respective isotopic differences as a measure of plant transpiration (Δ δD_leaf_ − δ^18^O_soil_) and soil evaporation (Δ δ^18^O_soil_ − δD_precipitation_) (Figs S8 and 9; see methods)^25^,^30^,^31^. Our stable isotope data span the time between 8.5 to 3.5 Ma, corresponding to the depositional time frame of the exceptionally well exposed Miocene Palo Pintado and Pliocene San Felipe formations of the AB.

Our isotope records distinguish between two stages in eco-hydrological and topographic conditions along the eastern AP: (1) a humid phase, which corresponds to a contiguous open foreland setting; and (2) an arid phase, representing an intermontane basin setting following the establishment of an orographic barrier (Sierra León Muerto) east of the present-day AB (Figs 1 and 2). Below we discuss the sequence of environmental changes and the corresponding regional tectonic forcing.

Paleoenvironmental and sedimentologic data prior to deposition of the Palo Pintado formation indicate arid conditions within the AB^32^,^33^. With the onset of deposition of the Palo Pintado formation between ca. 8.5 to 7.6 Ma δD_wax_ and δ^18^O_sc_ values decreased from ca. −110 to −150%, and 23 to 18%, respectively, indicating a trend towards wetter conditions in this inherently dry region (Fig. 2). For the same time interval the δ^13^C_wax_ and δ^13^C_sc_ records, pollen and plant macrofossil data suggest a stable C3 forest ecosystem in the AB (Fig. 3)^32^ likely similar to present-day environmental conditions in the foreland ca. 150 km to the East of the AB^22^. The isotopic composition of intercalated volcanic ash deposits (glass shards) within the AB section (δD_vg_) supports this notion as it indicates that meteoric water in the contiguous AB had a similar stable isotope composition at that time as modern meteoric waters from the undeformed foreland (Fig. 2)^21^. The wetter conditions indicated by the decrease in δD_wax_ and δ^18^O_sc_ values until 7.6 Ma may have resulted from enhanced orographic rainfall as a result of regional topographic growth along the Eastern Cordillera as inferred from low-temperature thermochronology (Sierra Luracatao, Cerro Durazno and Sierra de Quilmes)^8^,^32^,^34^,^35^. The evapotranspiration data (Fig. 3) suggest that the relative amounts of evaporation and transpiration were reduced in the AB in concert with enhanced orographic precipitation, whereas plant transpiration appears to have been unaffected (Fig. 3).

Between 7.6 and 6.5 Ma the sedimentation rate in the AB doubled from 0.5 to 1.0 km/Myr, and sedimentary environments changed from alluvial fans to meandering rivers with overbank deposits and paleosol formation (Fig. 2). Over the same time period both δD_wax_ and δ^18^O_sc_ records consistently display a large variability in isotope values (Fig. 2) of more than 50%, and 8%, respectively. These large variations in stable isotope values imply considerable changes in rainfall variability on timescales larger than each proxy record’s integration time. δD_wax_ and δ^18^O_sc_ integrate their isotopic signal over similar timespans, whereas δD_wax_ reflect soil formation timescales on the order of ca. 1 to 5 ka as a function of soil thickness^36^ and δ^18^O_sc_ represents integration time on the order of 0.1 to 10 ka^37^,^38^. Collectively, the isotope data point to the existence of a paleo LLJ south of 22°S as early as 7.6 Ma that facilitated moisture transport into the inherently dry region. Most likely the intensity was modulated by solar insolation linked to orbital variations reflected by the large variability in δD_wax_ and δ^18^O_sc_ values. The inference of a regionally extensive humid climate at that time is supported by the character of sedimentary facies, fossil content and isotope geochemistry of paleosols from other basins along the eastern flanks of the AP. These areas include basins close to the Argentina-Bolivia border at 22°53′S and 64°36′W^39^ and regions in southern Bolivia at 21°S^8^. Interestingly, the inferred onset of the LLJ in NW Argentina is about one million years later than its postulated impact in southern Bolivia^8^, suggesting a latitudinal lag time along the eastern Andes. The AB carbon isotope records of δ^13^C_wax_ and δ^13^C_sc_ during this period document little variation of 3.2% and 3.9%, respectively, reflecting a sustained C3 forest ecosystem (Fig. 3)^30^,^40^. This is corroborated by abundant tree trunks, leaf macrofossils, and pollen assemblages from the Palo Pintado Formation that resemble the present-day Paranaense flora of southwest Brazil^32^ (Fig. 2).

After approximately 6.5 Ma the variability in δD_wax_ and δ^18^O_sc_ values decreased to less than 38% and 1%, respectively. Prior to the decrease in variability δD_wax_ and δ^18^O_sc_ values increase over a short period of only 0.2 Myrs from −160 to −102% and from 18 to 31%, respectively. We interpret this signal to reflect the rapid establishment of arid conditions in the AB in the absence of regional circulation and moisture source changes at that time^8^,^10^. Instead, tectonic shortening and uplift at the eastern basin margin (the present-day Sierra de León Muerto) at ca. 7 Ma^35^ caused the formation of an effective orographic barrier and the transition from a contiguous foreland with E directed drainage into an intermontane basin setting with axial, N-S oriented drainage conditions (Figs 1 and 2)^32^. Today, the threshold elevation for effective orographic barriers blocking approximately 90% of incoming moisture in the south-central Andes is ca. 1.5 to 2 km^2^,^22^. Therefore, the increase in δD_wax_ and δ^18^O_sc_ values at 6.5 Ma can be interpreted to reflect rapid aridification in response to the Sierra de León Muerto reaching a threshold elevation of 2 km. This is compatible with a previous study that suggest that the coeval decrease in δD_vg_ values (Fig. 2) resulted from stronger Rayleigh rainout due to uplift^41^. Surface uplift along the eastern basin margin coincides with a rapid increase in plant transpiration in response to reduced moisture supply into the AB (Fig. 3). In contrast to the basin margin, the basin itself did not gain significant elevation during this initial stage of deformation and uplift, as indicated by a ca. 6 Ma caiman fossil (*Caiman latirostris*), whose extant relatives live below 800 m in the modern Andean foreland (Fig. 2)^32^,^42^. Subsequently, however, surface uplift of the intermontane basin involved strata within the AB and resulted in the deposition of coarser sediments of the San Felipe Formation (Figs 1 and 2)^35^. As a result of continued surface uplift (5.5 and 3.5 Ma) the basin further aridified as documented by overall increasing δD_wax_ values (ca. 130% to −98%), thick calcrete-bearing paleosols, and deposition of halite and gypsum in restricted fluvio-lacustrine environments (Fig. 2)^43^.

During this episode the ecosystem fundamentally changed as revealed by δ^13^C_wax_ and δ^13^C_sc_ values increasing from −32 to −28% and −11 to −8%, respectively (Fig. 3). These new environmental conditions could either correspond to a mixed C3/C4 high-altitude grassland ecosystem or represent greater water use efficiency of plants in an environment with low water availability, or both^6^,^44^. An environmental shift towards a C4 successor vegetation is also supported by rodent tooth enamel isotope data from the San Felipe Formation, which points to increased availability of C4 plants^43^. Reconstructed post-6 Ma clumped isotope temperatures indicate lower temperatures between 18° to 25 °C, similar to present-day summer temperatures^21^ and much cooler than previously inferred foreland temperatures of up to 38 °C at 7 Ma^17^. Uplift of the orographic barrier and transition to cooler, more arid conditions are reflected by sustained high levels of plant transpiration, evaporation, and evapotranspiration in the AB (Fig. 3). The large increase in leaf water enrichment of >60% inferred from terrestrial organic material during orographic barrier uplift is similar to the present-day difference between the Amazonian rainforest and the semi-arid basins along the eastern Andes (Figs 3 and S9)^25^.

In summary, our new multi-proxy, multi-isotope record from the AB unravels the intimate coupling of tectonic and climatic events in the southern central Andes and major Mio-Pliocene eco-hydrological changes that were part of a continent-wide redistribution of moisture, as predicted by General Circulation Models e.g.^4^,^10^. Our observations validate these models of enhanced convective rainfall and moisture transport via the LLJ in concert with progressive surface uplift of the AP^10^. The resulting seasonally hot and humid climate of the southern central Andes was characterized by variable amounts of evapotranspiration related to the strength of regional moisture transport via the LLJ (Fig. 3) and the establishment of a C3 forest ecosystem independent of global climate change. Our findings agree with climate models that point to a strong control of Andean topography on regional climate and a rather weak link to late Miocene - Pliocene global climate change e.g.^4^,^14^,^45^,^46^. Although decreasing atmospheric carbon dioxide levels, air and sea surface temperatures throughout the Late Miocene^47^,^48^,^49^ also impact deuterium and oxygen stable isotope values in South America^14^ the close proximity of our record to the uplifting eastern flanks of the AP dominates the climatic and eco-hydrologic signal in the AB deposits. This is remarkably well illustrated by the correlation of tectonic events and related environmental changes within the AB (Figs 2 and 3). However, humid eco-hydrologic conditions related to the orographic barrier of the AP flanks rapidly ended after 6.5 Ma as local orographic barrier uplift farther east forced cooler and drier conditions in the AB. As a result, we observe that stable isotope proxy records of plant wax and pedogenic carbonates primarily respond to thresholds of moisture supply in the lee of orographic barriers rather than surface uplift or regional to global climate change. The large eco-hydrologic gradients observed over time support the notion that mountain building forces landscape and climate change, and thus ecological gradients and physical habitats that set the stage for speciation and biodiversity^20^.

## Methods

**Figure 3:**
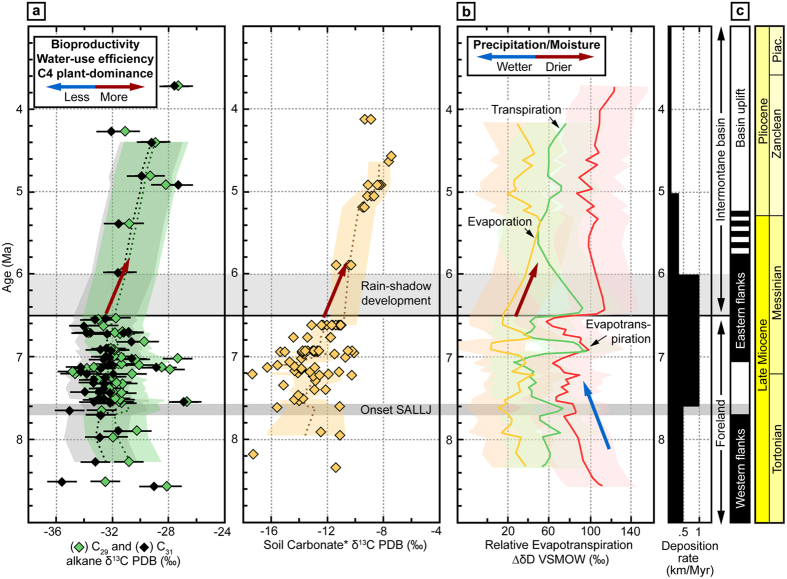
Carbon stable isotopes, vegetation and reconstructed evapotranspiration. (**a**) Stable isotope records of leaf wax δ^13^C_wax_ (this study) and soil carbonate δ^13^C_sc_ (this study and data from *Bywater-Reyes *et al*.^43^. Carbonate isotope records are reported according to the Pee Dee Belemnite (PDB). All isotope data has a 2-sigma age error of 0.1–0.3 Ma and δD_wax_ and δ^18^O_sc_ represent pooled precisions of 0.5% based on external isotope standards and analytical errors, respectively. (**b**) Reconstruction of evaporation, transpiration and evapotranspiration based on proxy source-water reconstruction. Reconstructed source-waters for lipid-biomarkers, pedogenic carbonates and volcanic glass shards have different source-waters for the individual proxies: volcanic glass - precipitation; pedogenic carbonates – soil water; lipid biomarkers – leaf water. For comparison δ^18^O_sc_ source waters are converted to δD values.

The multi-proxy-isotope approach presented here represents one of the first steps to reconstruct the different amounts of evaporation, transpiration and evapotranspiration from a single record on a million of year timescale. Assuming that individual δD_vg_ and δ^18^O_sc_ source waters are representative of precipitation and soil water, respectively, the isotopic difference between both values must then represent evaporation^30^,^31^ (Fig. S8). In addition, the difference between δ^18^O_sc_ and δD_wax_ source water values reflects leaf-water isotope enrichment above soil water, and thus transpiration^25^,^30^,^31^ (Fig. 3; Figs S8 and S9). Ultimately, the difference between reconstructed source-water values from δD_vg_ and δD_wax_ therefore represents evapotranspiration (Fig. 3; Figs S8 and S9). The relationships between the isotopically distinct water sources recorded by the proxies (leaf wax – leaf-water; soil carbonates – soil water; volcanic glass – meteoric water) and evaporation, transpiration and evapotranspiration are schematically sketched in Fig. S8. In combination the three isotope proxies can be used to reconstruct the total relative evapotranspiration trends in a region (Figs S8 and S9). To compare the data time series on a common timescale with the same temporal resolution we calculated equidistant time series based on a 100 ka time window. For calculating the equidistant time series we used MATLAB**^©^** (version R2010b) and used the method “interpl” with the specification “linear”. From the equidistant time series for leaf wax, soil carbonates and volcanic glass source waters we calculated the differences between each proxy representing evaporation, transpiration and evapotranspiration. The underlying individual source water reconstructions are discussed in further detail below.

δD_wax_ values are determined by the isotopic composition of water that is available for biosynthesis of leaf wax n-alkanes representing leaf-water and the net biosynthetic fractionation called ε_bio_ that accounts for various biochemical hydrogen isotope fractionations during biosynthesis of n-alkanes (Fig. S8). Assuming ε_bio_ is constant over the timescales of proxy integration in sediments, it is possible to reconstruct the isotopic composition of leaf-water from leaf-wax n-alkanes. The isotopic difference between the plants source water, i.e. soil-water, and leaf-water should thus arise due to (D)-enrichment in response to plant transpiration (Fig. S8)^25^. While εbio of leaf waxes from sedimentary deposits, integrating a vast number of plant individuals and species, can be regarded constant, recent studies reported a significant spread in biosynthetic fractionation among different plant species between ca. −130 to −190%^25^. For leaf-water reconstruction we use a combined ε_bio_ value of −160 ± 30% representing the range of reported ε_bio_ value for higher terrestrial plants from several available studies^27^,^50^,^51^. Reconstructed leaf-water is presented in Table S1 and Fig. S8 and source water differences in Fig. S9.

Pedogenic carbonates usually form at soil depths of ~20 to 30 cm as the result of evaporating surface water. As calcite forms it records a signal of δ^18^O and δ^13^C stable isotopes. The δ^18^O signal reflects the isotopic composition of the surface water and the δ^13^C signal has been interpreted to reflect bioproductivity, CO_2_ plant respiration or C_4_/C_3_ vegetation type. The calcite formation and the δ^18^O isotope values are strongly controlled by temperature-dependent isotope fractionation between water and carbonate^52^. Therefore, for the reconstruction of paleosurface water the carbonate formation temperature needs to be known. Here, we use reconstructed clumped-isotope temperatures from Carrapa *et al*.^17^ for deciphering the temperature-dependent isotope fractionation using the fractionation factor of Kim and O’Neil^17^,^52^. We linearly interpolate in between clumped-isotope temperatures points, where our sampling of soil-carbonates was denser. The error reported for reconstructed source water of δ^18^O_sc_ incorporates the analytical error for each analysis and was 0.1 to 0.3% (Table S2). In addition to analytical uncertainties other processes such as the location of pedogenic carbonate formation (e.g. open versus shaded for example) can have an effect on clumped-isotope temperatures of up to 10 °C that are used during reconstruction of source water^53^. Calculated values of the δ^18^O_carb_ source waters are reported in Table S2 and Figs 3 and S9. For comparison with δD_wax_ and δD_vg_ source waters the pedogenic carbonate δ^18^O_carb_ source water was converted to deuterium values using the present-day local meteoric water line of δD = 8.44*δ^18^O + 15.91 for 26°S from Rohrmann *et al*.^21^ (Table S2 and Figs 3 and S9)^21^.

After deposition rhyolitic glass incorporates large amounts of meteoric water (3 to 8 wt%) over a time frame of 5 to 10 ky^31^. The final δD_vg_ signal represents an integrated meteoric water signal during hydration over geological time scales. To compare δD_vg_ with other isotope proxy materials, e.g. lipid-biomarker and soil-carbonates, we converted the δD_vg_ signal to meteoric water δD values (δD_mw_), using the equation δD_mw_ = 1.0343 [1000 + δD_vg_ ] − 1000^31^. Converted meteoric-water and δD_vg_ values are presented in Table S3 and Figs 3 and S9.

## Additional Information

**How to cite this article**: Rohrmann, A. *et al*. Miocene orographic uplift forces rapid hydrological change in the southern central Andes. *Sci. Rep.* **6**, 35678; doi: 10.1038/srep35678 (2016).

## Supplementary Material

Supplementary Information

## Figures and Tables

**Figure 1:**
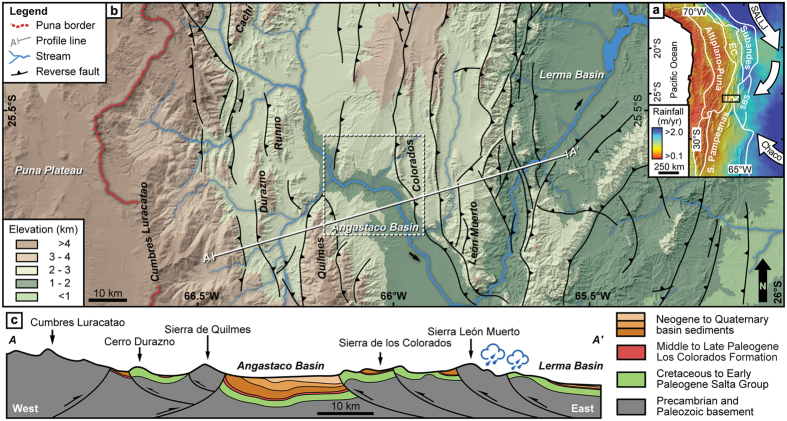
Rainfall and topographic patterns of the Central Andes and structural context of the study area. Figure 1 was created using Adobe Illustrator CS6 and the base map was created with ArcGIS 10.1. (**a**) TRMM 3B42 annual rainfall map and morphotectonic provinces (EC: Eastern Cordillera, SBS: Santa Barbara System)^2^. Arrows depict moisture transport direction (SALLJ - South American Low-Level Jet). (**b**) Topography and structural context of the south-central Andes and location of the study area. (**c**) Schematic geological cross section along profile line shown in B^33^,^34^. Cloud symbol indicates region of present-day enhanced orographic rainout, i.e. the position of the effective orographic barrier.

**Figure 2:**
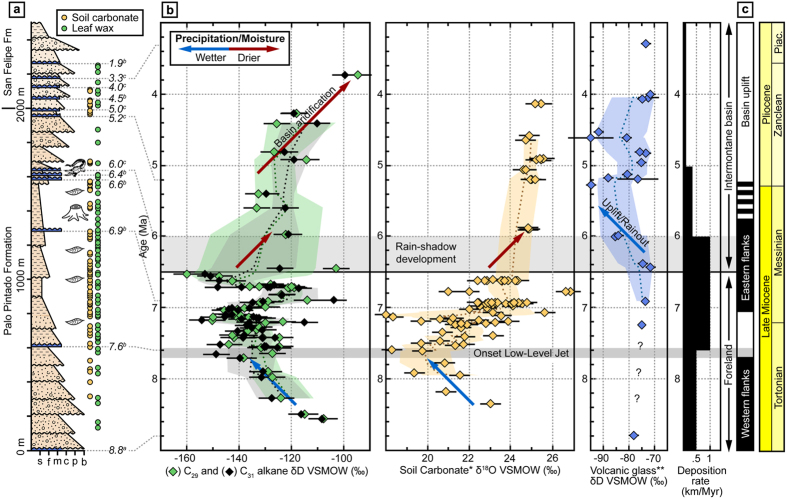
Stratigraphy, water stable isotopes, and tectono-sedimentary evolution of the Angastaco Basin. (**a**) Measured stratigraphic section showing fossil-bearing horizons (*Caiman latirostris*^42^ and fossil plant data from this study); Geochronological data from ^a^Carrapa *et al*.^35^, ^b^Pingel *et al*.^41^, and ^c^Bywater-Reyes *et al*.^43^; and position of leaf wax and soil carbonate samples. (**b**) Stable isotope compilation of leaf wax δD_wax_ (this study), soil carbonate δ^18^O_sc_ (this study and data from *Bywater-Reyes *et al*.^43^ and volcanic glass shards δD_vg_ Pingel *et al*.^41^. All isotope data has a 2-sigma age error of 0.1–0.3 Ma and δD_wax_ and δ^18^O_sc_ represent pooled precisions of 5% based on external isotope standards and analytical errors, respectively. δD and δ^18^O data are presented with respect to Vienna Standard Mean Ocean Water (VSMOW). (**c**) Deformation history based on own observations and low-temperature thermochronology^33^,^34^,^35^.
