# Supplementary material for: Miocene orographic uplift forces rapid hydrological change in the southern central Andes
Source: Sci Rep. 2016 Oct 21;6:35678. doi: 10.1038/srep35678 (PMC5073360; doi:10.1038/srep35678)
Supplement: Supplementary Information [file srep35678-s1.doc]

Supplementary Information: Miocene orographic uplift forces rapid hydrological change in the southern central Andes

**Alexander Rohrmann^1^,*, Dirk Sachse^1,2^, Andreas Mulch^3,4^, Heiko Pingel^1^, Stefanie Tofelde^1^, Ricardo N. Alonso^5^, Manfred R. Strecker^1^**

^1^Institut für Erd- und Umweltwissenschaften, Universität Potsdam, 14476 Potsdam, Germany.

^2^ GFZ German Research Centre for Geosciences, Section 5.1: Geomorphology, Telegrafenberg, 14473 Potsdam, Germany.

^3^Senckenberg Biodiversity and Climate Research Centre (BiK-F), 60325 Frankfurt/Main, Germany.

^4^Institut für Geowissenschaften, Goethe Universität Frankfurt, 60438 Frankfurt/Main, Germany.

^5^Departamento de Geología, Universidad Nacíonal de Salta, CONICET, 4400 Salta, Argentina.

*Correspondence to: rohrmann@geo.uni-potsdam.de

**Sampling and stratigraphic profile logging.** Samples for leaf-wax and pedogenic carbonate oxygen, hydrogen, and carbon stable isotope analyses were collected during two field seasons in 2011 and 2013 from the sedimentary basin record of the Angastaco Basin (~25.5°S). Sampling and section measurements (thickness and bedding orientation) were performed along an E-W transect in exposed successions of the Mio-Pliocene Angastaco, Palo Pintado, and San Felipe formations along the Río Calchaquíes near the town of Santa Rosa (Fig. S1). We focused our attention on organic-rich layers of the Angastaco basin strata with abundant paleosoils and lacustrine sediments intercalated with volcanic ash deposits. Overall, 66 leaf-wax samples and 64 pedogenic carbonate samples were collected from paleosoils, overbank deposits and lacustrine sediments. Our aim to collect equidistant samples was limited by suitable organic-rich material or soil-carbonate horizons for analysis. However, whenever possible, well-consolidated nodules were sampled at least 30 cm below paleosoil horizons. Sampling intervals for leaf-wax samples vary between 20 to 150 m, with higher sampling rates in sections with evidence for more humid depositional environments. Samples collected north and south of the measured stratigraphic profile were projected into the stratigraphic log, as individual beds can be traced along strike on satellite imagery.

**Sample preparation for leaf-wax δD_wax_ and δ^13^C_wax_ analyses and measurement.** Samples were crushed using DCM-cleaned (dichloromethane) equipment and pulverized (ca. 40 to 60 µm) in a shatterbox with agate grinding chamber. Soluble organic matter was extracted from samples (100 g) at the University of Potsdam using an accelerated solvent extractor (ASE350, Dionex Crop., Sunnyvale, USA) with a dichloromethane/methanol mixture of 9:1 at 100°C and 1500 psi. Total extracts of three 18-minute cycles were captured in 250 ml bottles, later concentrated to 4 ml in a Turbovap, and then separated on silica gel using a solid phase extraction (SPE). SPE-columns preparation included the use of 1.5 g of silica gel (0.040-0.063 mesh; Alfa Aesar, Ward Hill, USA) filled into 6 ml glass columns (Macherey-Nagel, Düren, Germany). Columns were cleaned with three times the column volume of acetone and DCM and then dried overnight at 60°C. The column was again flushed with three times the column volume of acetone, DCM, and hexane prior to transferal of the total lipid extract onto the column. n-Alkanes and alcohols were eluted in 15 ml hexane and DCM, respectively, and the remaining substances were flushed with 15 ml methanol. Two out of three separated fractions were stored for later analysis. The remaining n-alkane fraction was treated with 6 μg 5α-androstane standard for gas chromatographic quantification. The identification and quantification of individual compounds was performed using a gas chromatograph with a coupled flame ionization and mass-selective detector (GC-FID/MSD Agilent 7890A GC, 5975C MSD, Agilent Technologies, Palo Alto, USA) flushed with helium carrier gas. Temperatures in the GC oven were programmed to increase at a rate of 12°C/min starting from 70°C to 320°C at which temperatures were held constant for 21 min. The PTV injector had a split ratio of 5:1 at an initial temperature of 70°C. The injector was heated up to 300°C at a programmed rate of 7.2°C/min and held constant at this temperature for 2.5 min. The n-alkane FID-peak areas were compared with the previously added 5α-androstane standard from which n-alkane concentrations were calculated. The n-alkane concentrations are reported as μg per gram dry sediment. In addition the average chain-length (ACL) and the carbon preference index (CPI) were calculated in Mathlab®.

For all samples δD_wax_ and δ^13^C_wax_ were measured using a coupled gas chromatography-isotope ratio mass spectrometer (GC-IRMS) Delta V Advantage (ThermoFisher Bremen, Germany) at the University of Potsdam. The n-alkane fractions were dried and concentrated to 200 μg/μl per compound in hexane for δD measurements. The n-alkane fraction was injected (1 μl) into an TRACE 1310 Gas Chromatograph equipped with an Agilent DB-5 column, 30 m x 0.25 mm x 25 μm film. The injector was operated in splitless mode at 300°C and the oven was held at 70°C for 2 min. The oven was heated at 15°C/min until 150°C, and then heated with 5°C/min to 320°C. The final temperature was held for 10 min. The column effluent was transferred via a ConFlo IV interface (ThermoFisher, Bremen, Germany) into an isotope ratio mass spectrometer after conversion to H_2_ in a high-temperature oven at 1420 °C. In general, triplicate analysis of 25 samples was performed, however, for 31 samples we performed only duplicate analysis. All results are reported using the conventional delta notation in permil (‰) units. δD values were corrected using a calibrated and known standard mixture of n-C_16_ to n-C_30_ alkanes obtained from the Biogeochemical Laboratories of the University of Indiana (A_3_, A_4_ and A_5_) as well as a concentration depended standard mixture (B_2_, B_3_). A Linear regression was produced using the known vs. measured values of the A_4_ and A_5_ standards, having a linear regression slope of 1 ± 0.021 for all analyzed standards. Three standards were measured after every sixth to ninth injection, where an A_3-5_ standard was measured in the beginning and at the end and a B_2_ standard in the middle of each sequence. The H_3_^+^ factor was determined at the start and end of the sequence and was very robust over the measurement period at 4.4 ± 0.25 (*31*). The analytical standard deviation for all single measurements was typical better than ± 3‰ (Table S1). We report averaged sample duplicate and triplicate measurements with a standard deviation of ± 5‰ that represents the total variability in all measured n-alkane standard mixtures of the A_3-5_ standards and is more than the analytical standard deviation of each single measurement of ± 3‰.

δ^13^C_wax_ values were measured using the same fractions used for δD_wax_. The n-alkane fractions were concentrated to 60 μg/μl in hexane for δ^13^C measurements. The same instrumental setup (GC-IRMS) and temperature programming was used as for the δ^13^C measurements, only the oxidation oven was run at a lower temperature of 960°C. Duplicates were measured for each sample and a CO_2_ gas with known isotopic composition was used as reference gas. The same n-alkane standard mixtures (A_3-5_ and B_2_ standard) were used as for δD measurements with the same standard setup in the measured sequence. Only duplicate analysis for each sample was performed. The standards were used to correct the analyzed samples to Vienna Standard Pee Dee Belemnite scale (VPDB). A Linear regression was produced using the known vs. measured values of the A_4_ and A_5_ standards and linear regression had a slope of 1 ± 0.14 for all analyzed standards. The results are reported in delta notation in permil (‰). The analytical precision of each single measurement had a typical standard deviation of ± 0.5‰. We report all measured samples with a standard deviation of ±1‰, which represents the total variability in all measured n-alkane standard mixtures of the A_3-5_ standards and is more than the analytical standard deviation of ± 0.5‰. All lipid-biomarker n-alkane data of δD and δ^13^C and ACL, CPI and cross correlation plots are reported in Table S1; Figs. S2-S7 and Figure 2 and 3.

**Sample preparation for soil-carbonates δ^18^O and δ^13^C analyses and measurement.** In total 64 pedogenic carbonates were selected from the Angastaco Basin, ranging from ~10 to 4 Ma. Pedogenic carbonate nodules were cut in half and bulk carbonate powder was extracted with a diamond tip dental drill. 100 to 180 μg untreated pedogenic carbonate was reacted with 98% H_3_PO_4_ for 90 min at 70°C in continuous flow mode using a Thermo MAT 253 mass spectrometer interfaced to a Thermo GasBench II at the Goethe University-BiK-F Joint Stable Isotope Facility Frankfurt. Analytical precision was typically ±0.1‰ for δ^18^O and ±0.2‰ for δ^13^C based on replicate measurements of international and in-house standards. Stable isotope values are presented with respect to VSMOW (δ^18^O) and PDB (δ^13^C). All pedogenic carbonates δ^18^O and δ^13^C values are reported in Table S2 and Figure 2 and 3.

**References**

A. W. Hilkert, C. B. Douthitt, H. J. Schluetter, W. A. Brandt, Isotope ratio monitoring gas chromatography / mass spectrometry of D / H by high temperature conversion isotope ratio mass spectrometry. *Rapid Commun. mass Spectrom.* **13**, 1226–1230 (1999).


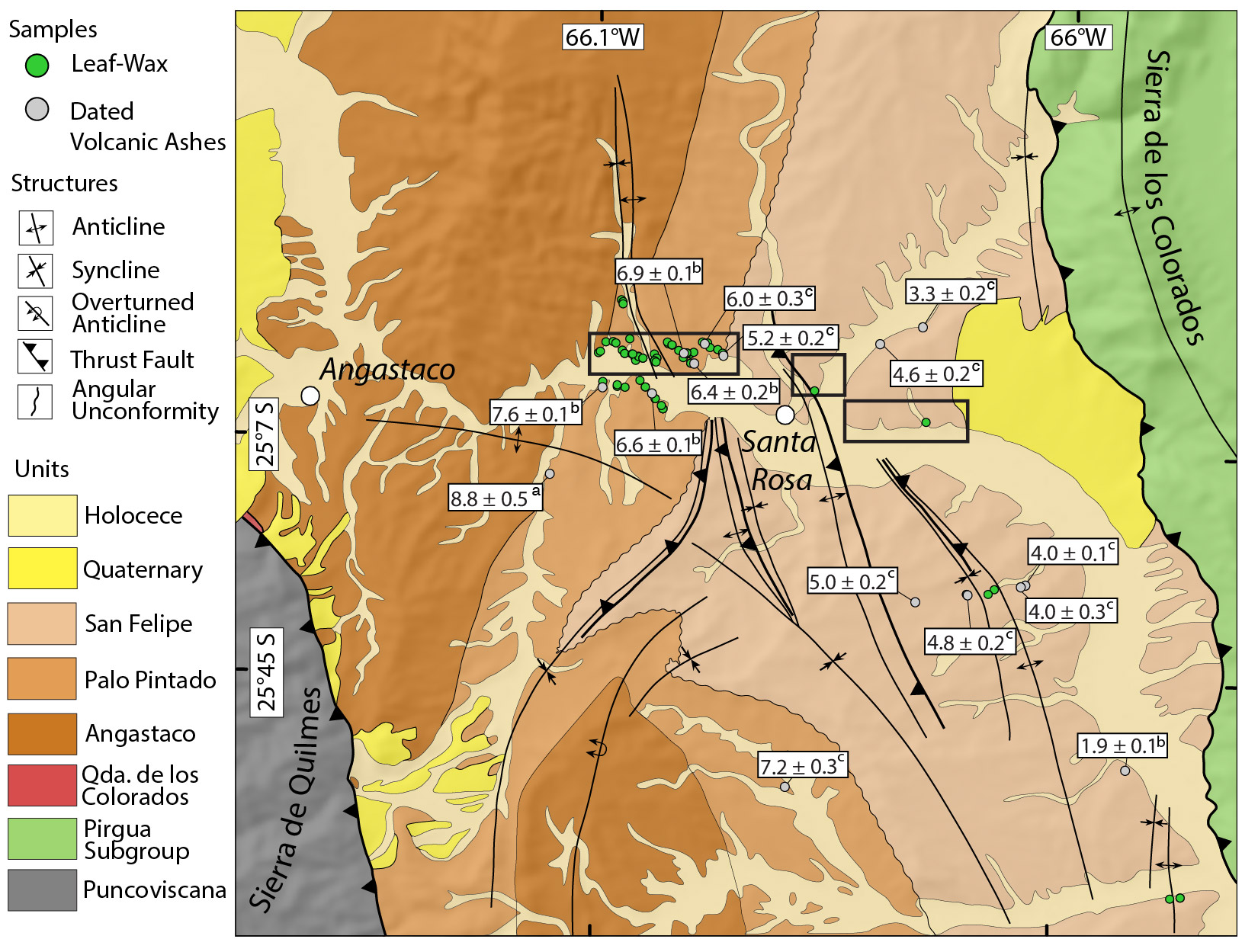


**Figure S1** Regional tectonic overview of the study area and stratigraphy. Figure 1 was created using Adobe Illustrator CS6 (http://www.adobe.com/products/illustrator.html) and the base map was created with ArcGIS 10.1 (http://www.esri.com/software/arcgis). Geological map of the Angastaco Basin (*22*) with leaf-wax sample locations and volcanic ash dates (for soil carbonate sample locations see Table S2). See legend for symbols and colors. Black boxes mark location of the measured stratigraphic log presented in figure 2. Shown U-Pb zircon ages of volcanic ashes are from: ^a^Carrapa et al. (2011), ^b^Pingel et al. (2016), ^c^Bywater-Reyes et al. (2010) (*22, 25, 27*).


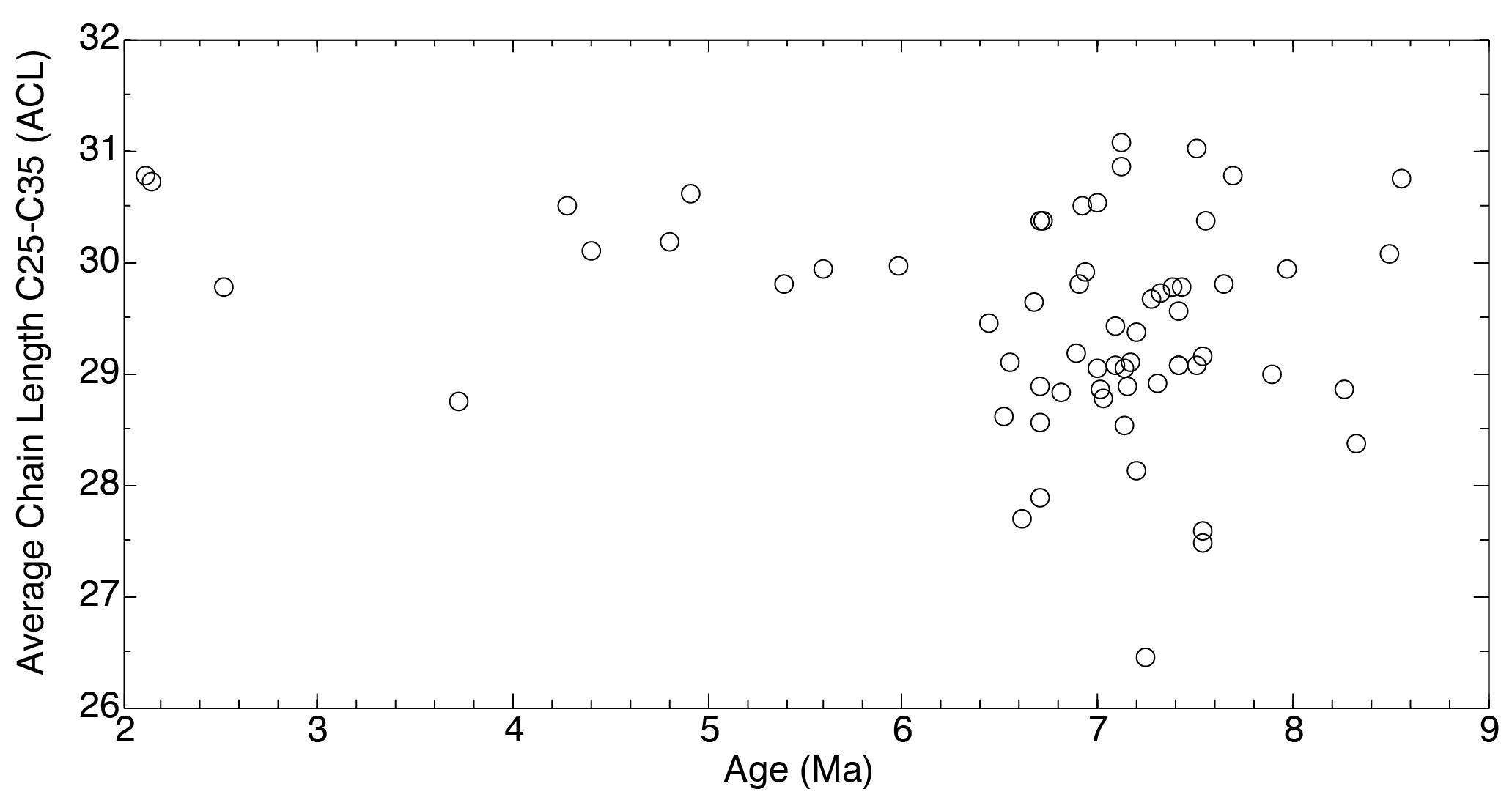


**Figure S2** Average chain lengths (ACL) for n-alkane records C_25_ to C_35_.

**
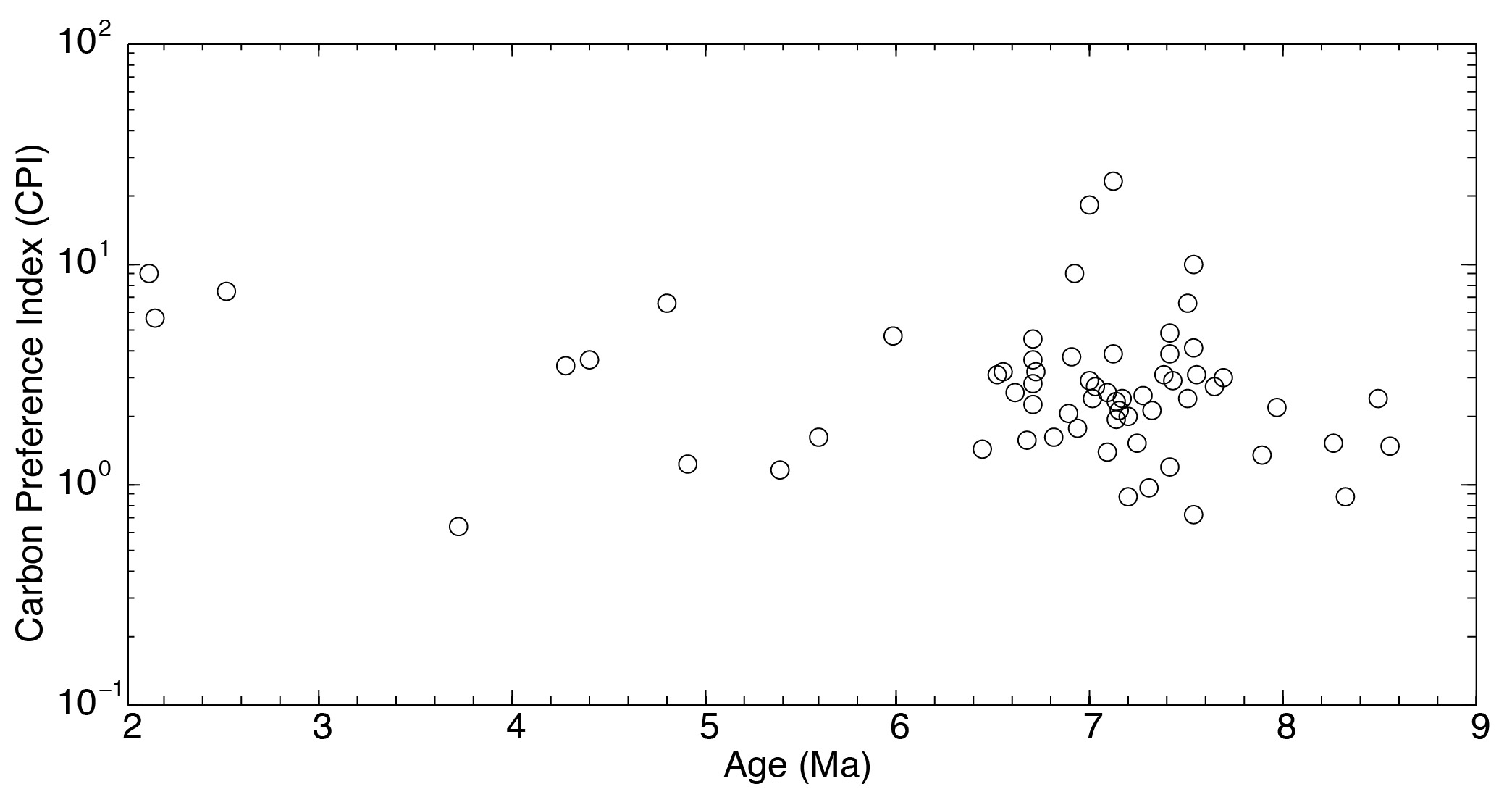
**

**Figure S3** Carbon preference index (CPI) for n-alkane records.

**
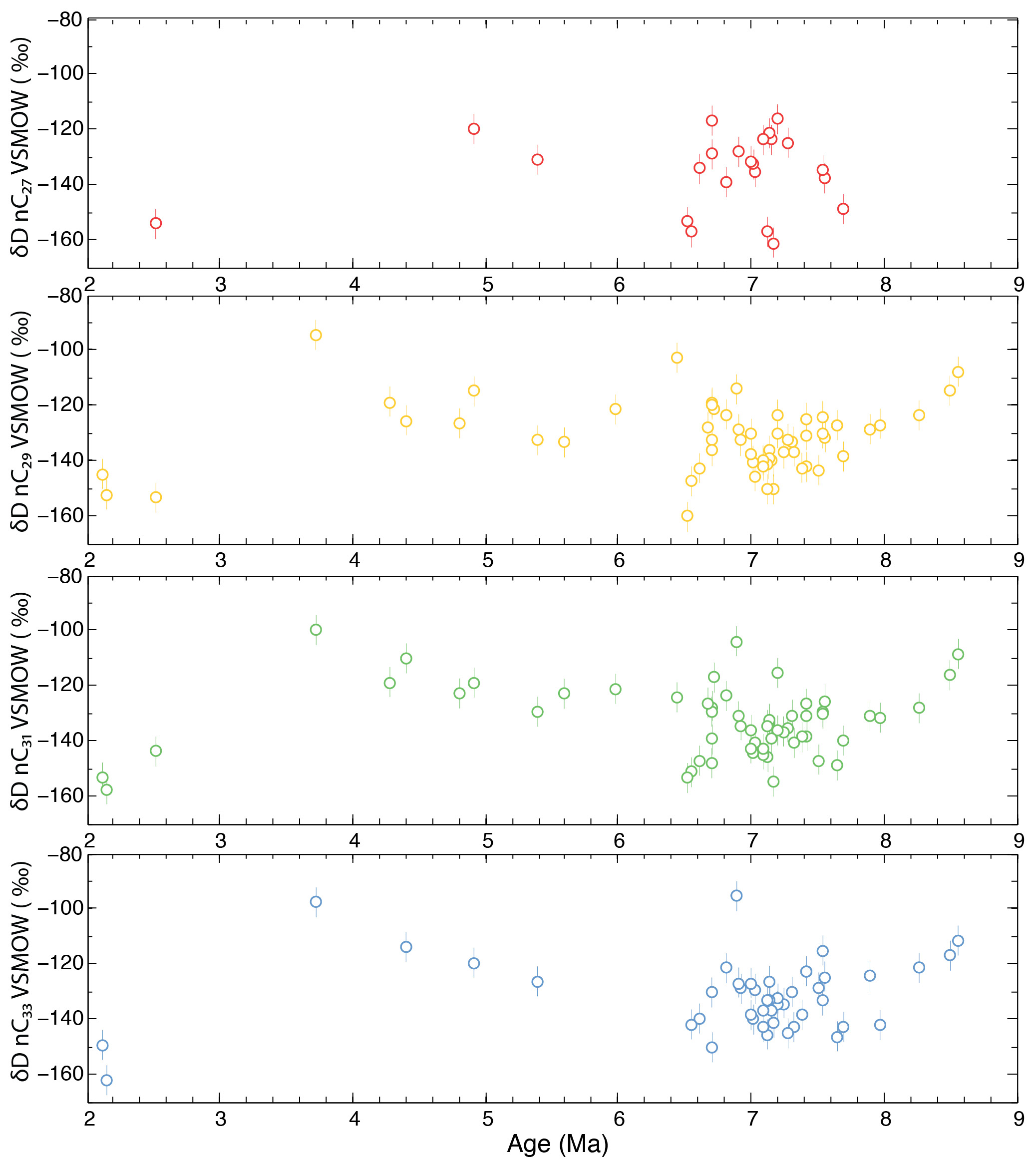
**

**Figure S4** Hydrogen lipid biomarker data for n-alkanes C_27_ to C_33_ versus age.

**
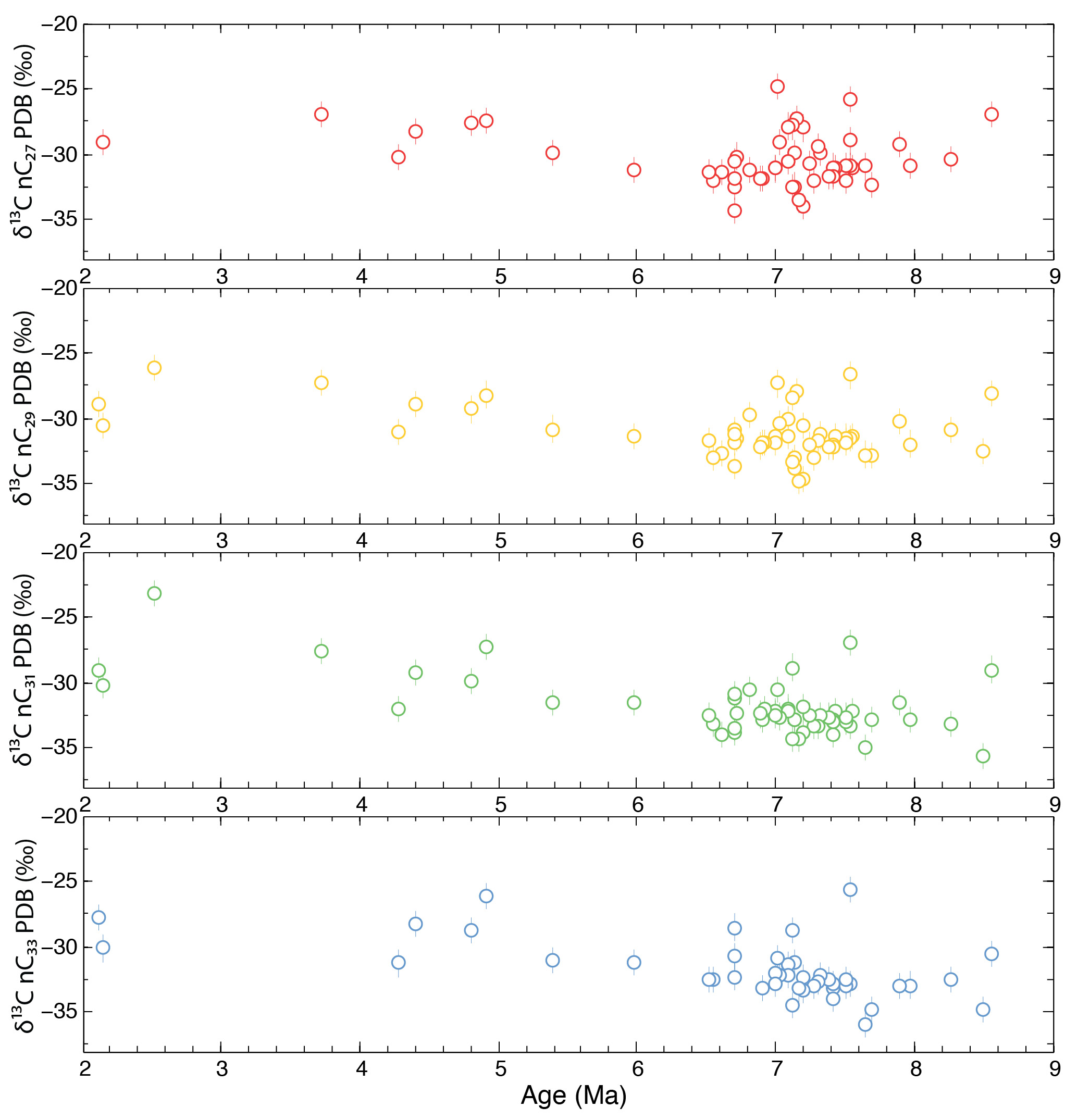
**

**Figure S5** Carbon lipid biomarker data for n-alkanes C_27_ to C_33_ versus age.


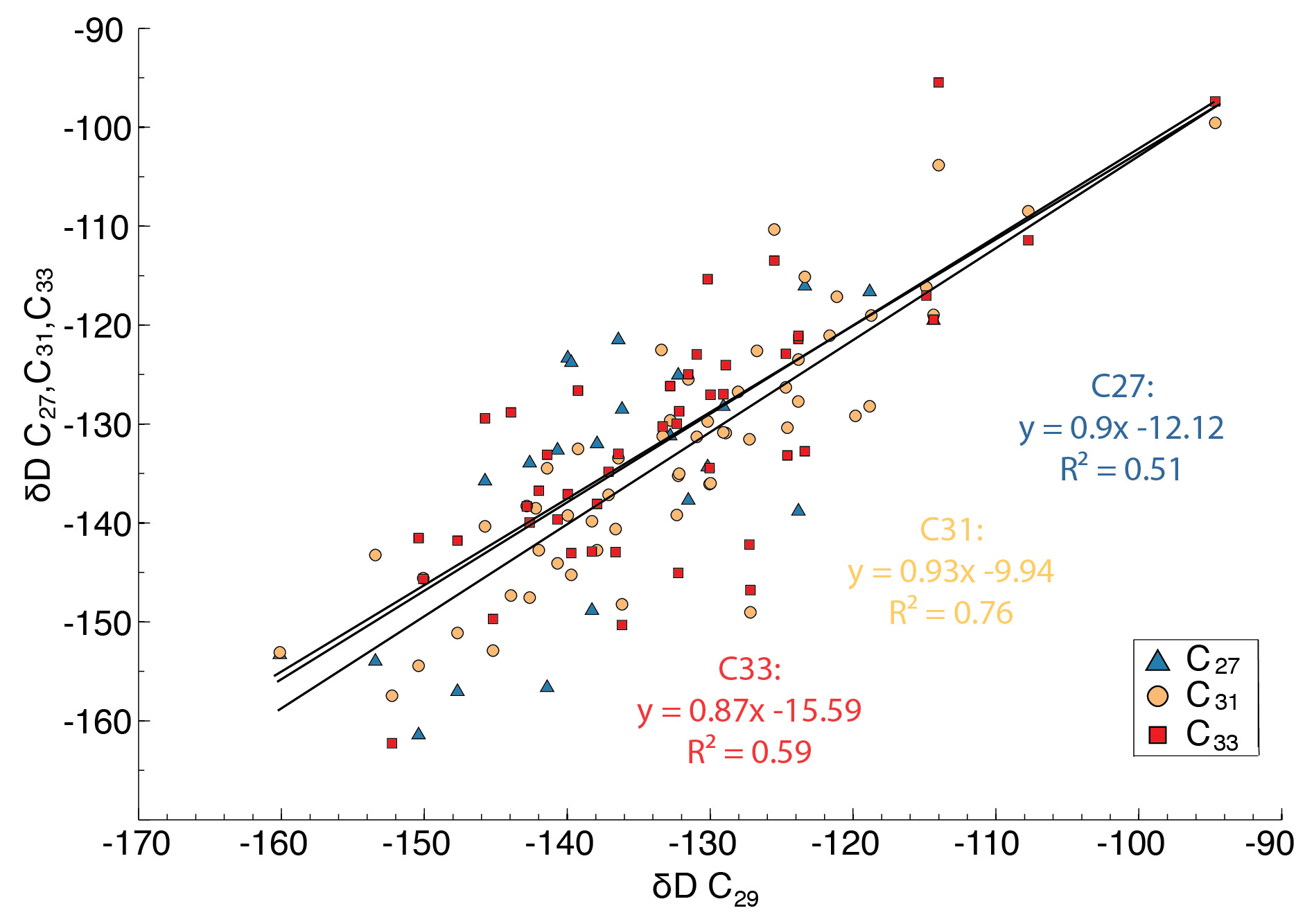


**Figure S6** Plot of hydrogen for C_29_ versus C_27_, C_31_ and C_33_ to decipher hydrogen sources and plant groups (e.g., higher plants, aquatic plants, grasses).


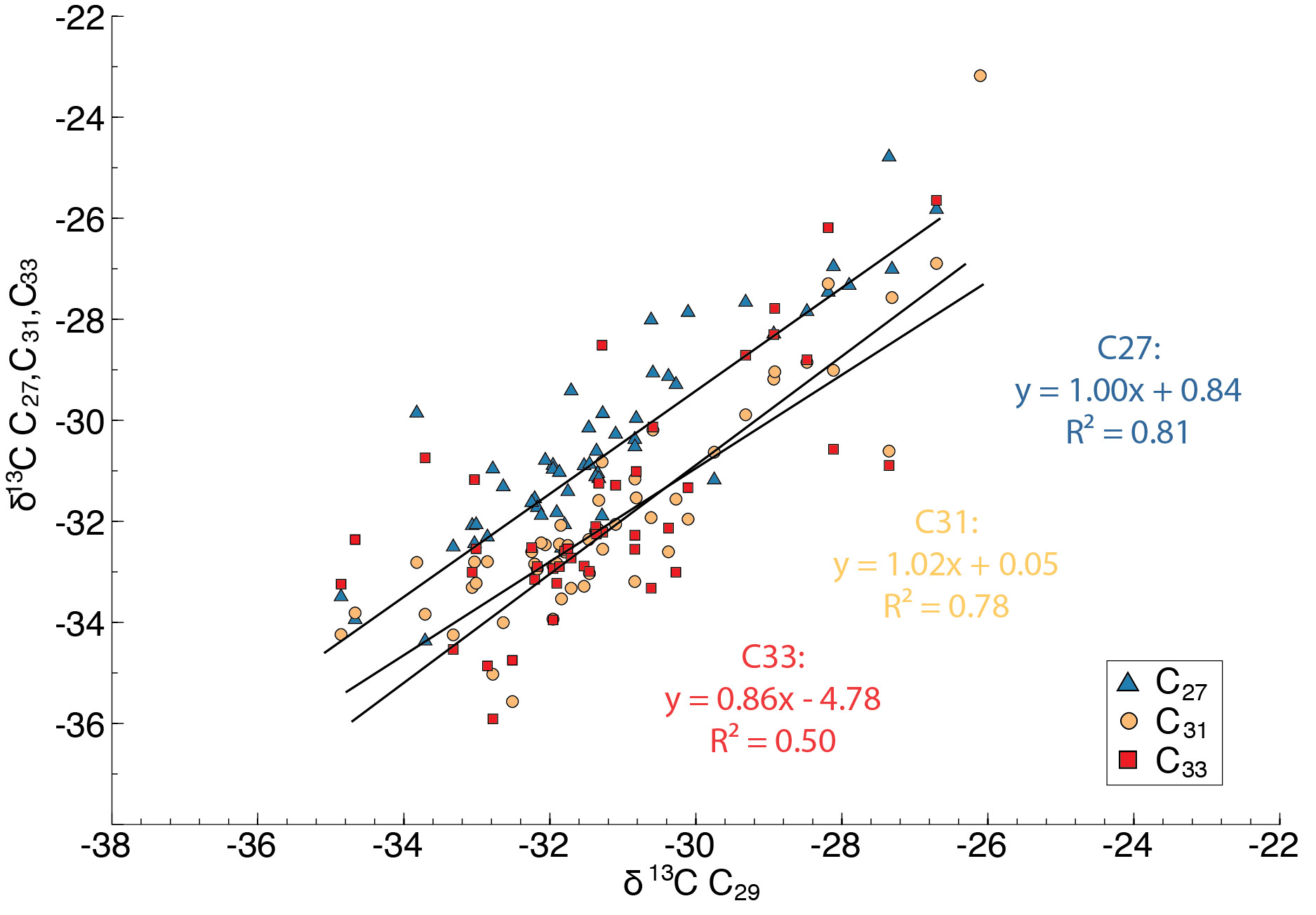


**Figure S7** Plot of carbon C_29_ versus C_27_, C_31_ and C_33_ to decipher carbon sources and plant groups.


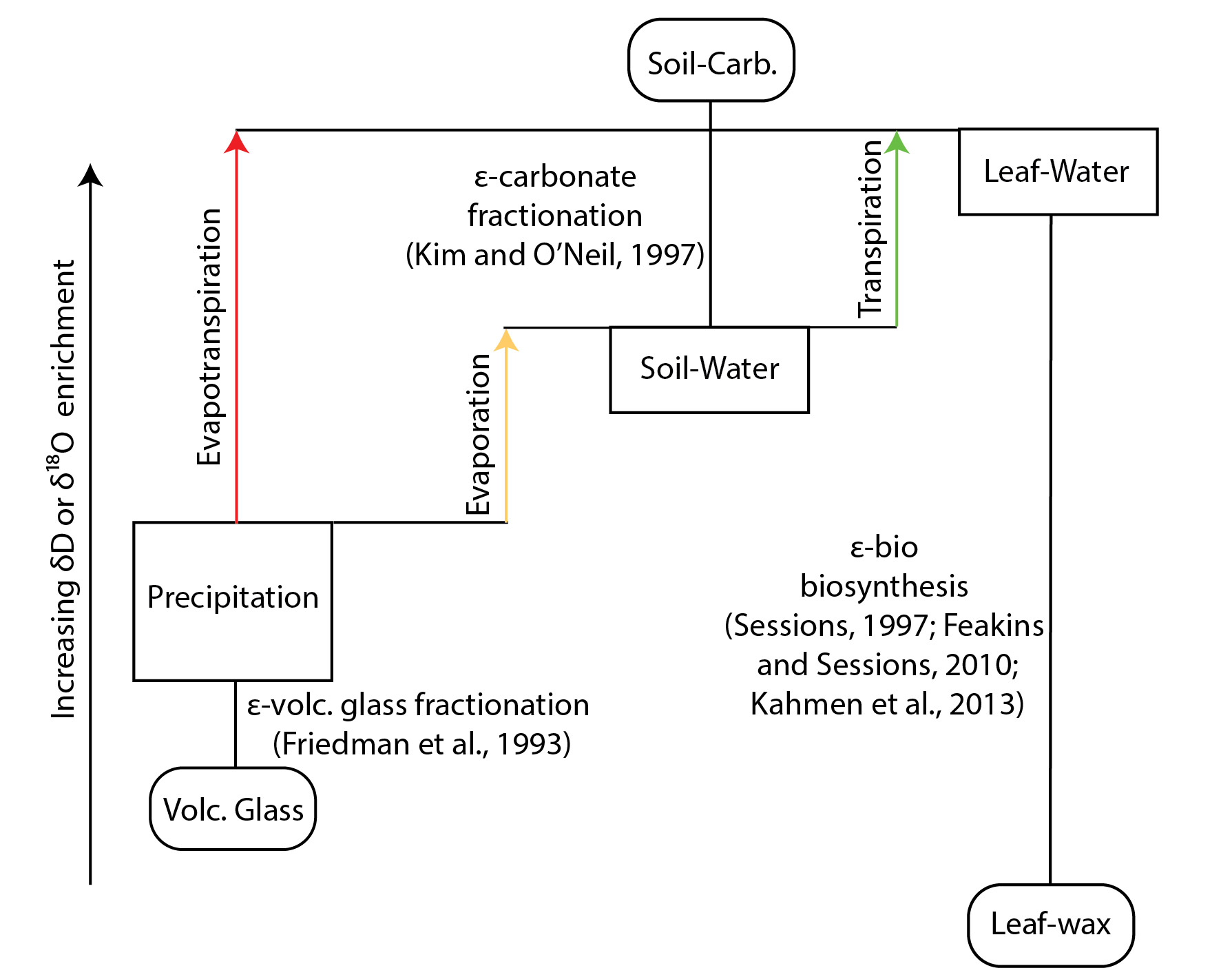


**Figure S8** Isotopic relationships between source water δD and δ^18^O of precipitation, soil-water and leaf-water and stable isotope proxies of lipid biomarkers, soil-carbonates and volcanic glass. The figure gives information about the recorded water source in each proxy, e.g. leaf-wax records leaf-water and corresponding fractionation factor. The figure is structure that increasing δD or δ^18^0 values in each proxy or source water are towards the top of the figure (arrow). The potential arising differences between the different source waters are thus interpreted to reflect transpiration, evaporation and evapotranspiration.


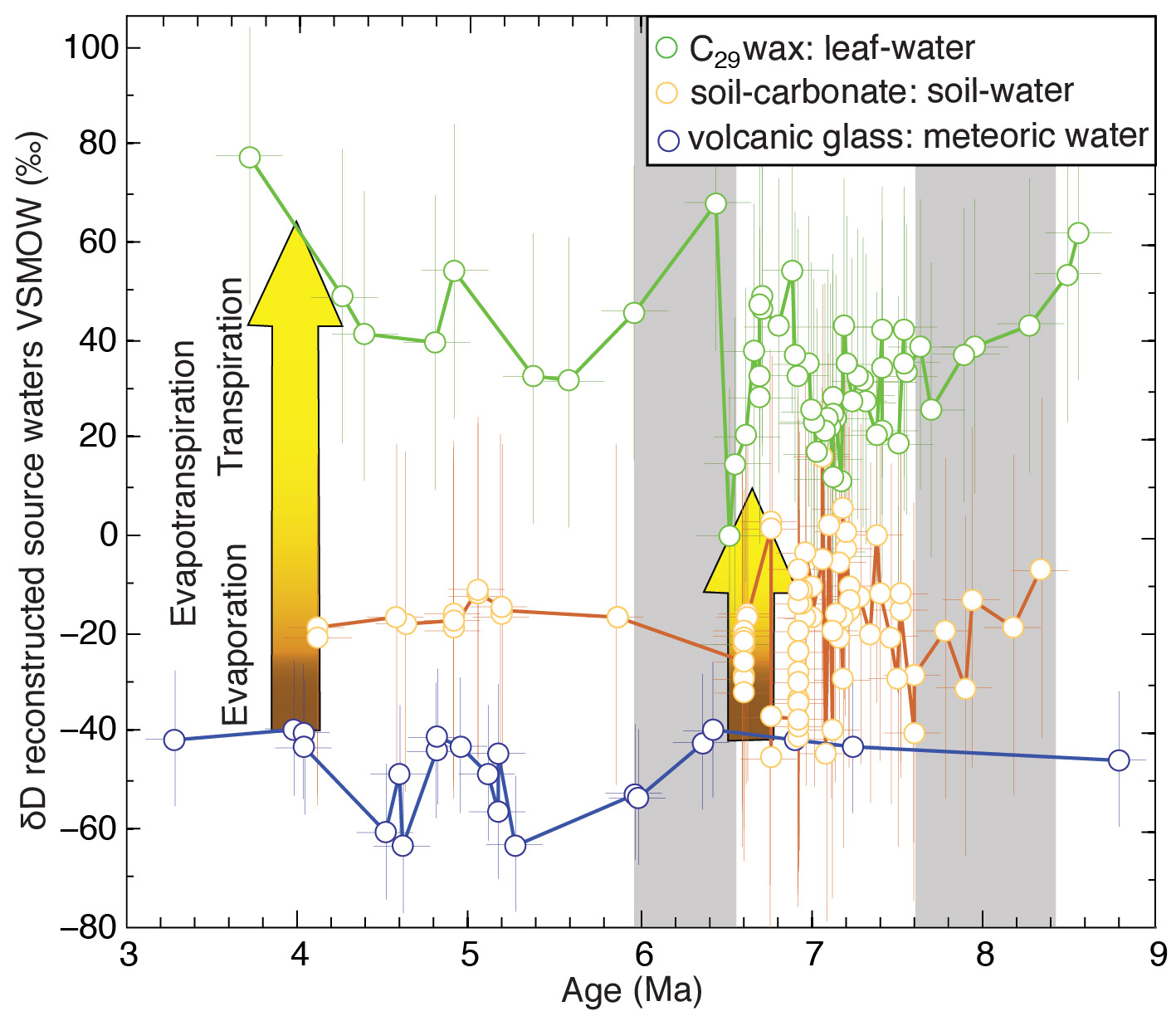


**Figure S9** Reconstructed source water for lipid biomarkers, pedogenic carbonates, and volcanic glass. Reconstructed source water and estimates of evaporation, transpiration, and evapotranspiration over time (arrows). Note the differences in reconstructed source water for the different proxies, suggesting that all three proxies use different source water: volcanic glass: precipitation, pedogenic carbonates: soil water, lipid biomarkers: leaf /soil water.


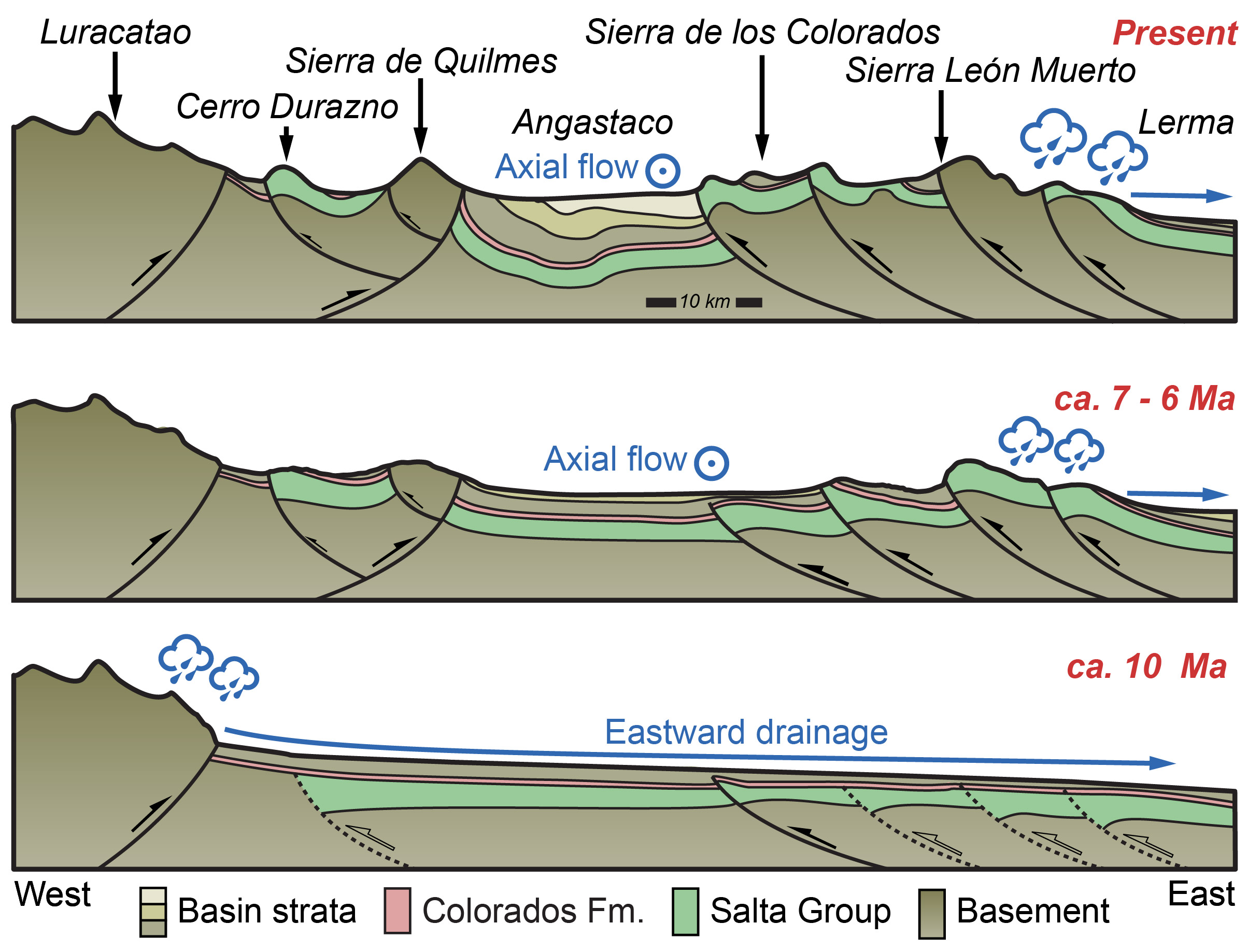


**Figure S10** Schematic geological cross section and reconstructed geologic history along profile line shown in Fig. 1B. Figure S10 was created using Adobe Illustrator CS6. The major compressional basins and ranges are shown (*20-21*). Cloud symbols indicate region of past and present-day enhanced orographic rainout, i.e. the location of an effective orographic barrier.

**Table S1** Leaf-wax n-alkane results and source water reconstruction

**Table S2** Pedogenic-carbonate results and source water reconstruction

**Table S3** Volcanic glass and source water reconstruction
